# Beyond Culture: Real-Time PCR Performance in Detecting Causative Pathogens and Key Antibiotic Resistance Genes in Hospital-Acquired Pneumonia

**DOI:** 10.3390/antibiotics14090937

**Published:** 2025-09-17

**Authors:** Lana Hani Abu Khadija, Shatha M. Alomari, Ahmad R. Alsayed, Heba A. Khader, Andi Dian Permana, Luai Z. Hasoun, Manar Saleh Zraikat, Walaa Ashran, Malek Zihlif

**Affiliations:** 1 Department of Pharmacology, School of Medicine, The University of Jordan, Amman 11942, Jordan; 2 Department of Pharmaceutical Science, Zarqa University, Zarqa 13110, Jordan; 3 Department of Clinical Pharmacy and Therapeutics, Faculty of Pharmacy, Applied Science Private University, Amman 11931, Jordan; 4 Department of Clinical Pharmacy and Pharmacy Practice, Faculty of Pharmaceutical Sciences, The Hashemite University, Zarqa 13133, Jordan; 5 Faculty of Pharmacy, Hasanuddin University, Makassar 90245, Indonesia; 6 Department of Surgical Critical Care, The University of Jordan Hospital, The University of Jordan, Amman 11942, Jordan

**Keywords:** antibiotic resistance, antimicrobial susceptibility, hospital-acquired pneumonia, molecular epidemiology, nosocomial infection, real-time PCR, ventilator-associated pneumonia

## Abstract

**Introduction:** The rise in hospital-acquired pneumonia (HAP) due to antibiotic-resistant bacteria is increasing morbidity, mortality, and inappropriate empirical antibiotic use. This prospective research aimed to evaluate the performance of a real-time polymerase chain reaction (PCR) assay for detecting causative microorganisms and antibiotic-resistance genes from respiratory specimens compared to traditional methods. Additionally, we aimed to determine the molecular epidemiology of antibiotic resistance genes among HAP patients at The University of Jordan hospital. **Methods:** Lower respiratory tract samples were collected from HAP patients, including those with ventilator-associated pneumonia (VAP), between May 2024 and October 2024. Clinical data from the medical files were used to collect and analyze demographic and clinical information, including clinical outcomes. Real-time PCR was run to detect causative microbes and antibiotic resistance genes. **Results:** Among 83 HAP patients (median age 63, 61.45% male), 48.15% died. Culture identified *Klebsiella* (25.53%), *Acinetobacter* (22.34%), and *Candida* (24.47%) as the most common pathogens, while qPCR showed higher detection rates, including for *A. baumannii* (62.20%, *p* = 0.02) and *K. pneumoniae* (45.12%, *p* < 0.001). Carbapenem resistance was high; *A. baumannii* showed 100% resistance to most antibiotics except colistin (92.31%). The resistance genes *ndm* (60%) and *oxa-48* (58.46%) were frequently detected and significantly associated with phenotypic resistance (*p* < 0.001). The qPCR identified resistance genes in all carbapenem-resistant cases. No gene significantly predicted mortality. **Conclusions:** Real-time PCR diagnostic technique combined with epidemiology of antibiotic resistance genes data may be a rapid and effective tool to improve HAP management. Large, multicenter studies are needed in the future to validate the performance of real-time PCR in HAP diagnosis, and appropriate management is also required.

## 1. Introduction

Pneumonia is an infection with characteristic inflammation of the lung alveoli [1]. The symptoms of nosocomial pneumonia, also known as hospital-acquired pneumonia (HAP), develop after at least two days of hospital admission. Approximately 13–18% of nosocomial infections are caused by HAP. Globally, HAP occurs in about 5 to 10 adults per 1000 hospitalization cases per year [2]. Also, ventilator-associated pneumonia (VAP) affects 10–25% of all mechanically ventilated patients. Among hospital infections, VAP is the leading cause of infection and death in the intensive care unit (ICU) [1]. Whereas HAP is ranked as the second most prevalent infection after urinary tract infection [2].

HAP and VAP are considered severe nosocomial infections with high morbidity and mortality impact [3]. Additionally, nosocomial pneumonia is among the top 5 conditions that result in increased medical cost due to higher rate of hospitalization [4]. VAP is often complicated by the development of sepsis and multiple organ failure, which lead to death [5].

Antimicrobial resistance, according to the definition of the Centers for Disease Control and Prevention (CDC), is an “urgent global public health threat, killing at least 1.27 million people worldwide and associated with nearly 5 million deaths in 2019”. Extended use of antibiotics commonly promotes the growth of resistance bacteria toward different antibiotics and suppresses other susceptible bacteria. This resistance is shown not only toward the currently used antibiotics, but also to other antibiotics from the same group [6]. Other potential side effects from continuous use of antibiotics include severe infections (e.g., Clostridioides difficile), thrombocytopenia, allergies, cardiovascular, and renal complications [7].

Because of the recent antibiotic resistance outbreak, management of HAP has become a worldwide challenge. A crucial strategy to overcome this issue is the use of targeted therapy after microbial diagnosis. Immediate detection of the causative microbe in addition to the related prevalent resistance genes is vital in respiratory infection management to avoid the unnecessary use of broad-spectrum antimicrobial agents. Various traditional diagnostic methods are used, such as conventional culture, antigen surveillance, and microscopic assessment. These methods have many drawbacks, as they have low sensitivity and long required procedures which usually need several samples to be collected. Lately, many types of polymerase chain reaction (PCR)-based tests have been designed to diagnose respiratory pathogens [8].

This study aims to evaluate the performance of real-time PCR assay for detecting causative microorganisms and antibiotic resistance genes from respiratory specimens compared to traditional methods. Also, we aimed to determine the molecular epidemiology of antibiotic resistance genes among HAP patients at The University of Jordan hospital.

## 2. Results

### 2.1. Demographic and Clinical Features

Our analysis includes 83 subjects diagnosed with HAP. A comprehensive examination of demographic information and baseline clinical characteristics was conducted. The findings from this analysis are summarized in Table 1.

According to the demographic profile of the study participants, the median age of the patients was 63 (IQR = 36.5), with a gender distribution of 38.55% female (*n* = 32) and 61.45% male (*n* = 51). Cardiovascular disease was most prevalent (57.83%, n = 48), followed by diabetes mellitus (38.56%, *n* = 32) and malignancies (24.10%, *n* = 20). Chronic kidney disease (CKD) and other lung diseases were observed in 19 out of 83 patients (22.89%). Regarding risk factors for HAP and/or VAP, neurological disorders, including stroke, showed the highest risk among the participants (36.14%, n = 30). Antibiotic use within three months (33.73%, *n* = 28), mechanical ventilation (32.53%, *n* = 27), tube feeding (31.33%, *n* = 26), and supine positioning of the patient (28.92%, *n* = 24) were significant contributors. Hyperglycemia and aspiration were less than 10% prevalent. The initial finding of HAP was most often seen on chest radiography, specifically lobar infiltration, which was seen in 72.29 percent of the participants. Tachycardia and altered breath sound were found in 30.12% (*n* = 25) and 26.51% (*n* = 22) of the patients, respectively. Symptoms such as cough, sputum production and SOB affected approximately 25% of participants. Also, tachypnoea was notable in 21.69% (*n* = 18) of the participants.

### 2.2. Clinical Data and Outcomes

The analysis of clinical data about the participants throughout their hospitalization is summarized in Table 2. The results showed that the median number of previous hospitalizations among patients was five (IQR: 6). Of the total cases, 31 patients (37.35%) were admitted to the general ward, while 52 (62.65%) required ICU care. The median ICU length of stay was 19.5 days (IQR: 22.75), while the overall hospital LOS was 25 days (IQR: 28). Regarding pneumonia classification, 60 patients (72.29%) were diagnosed with hospital-acquired pneumonia (HAP), and 23 (27.71%) had ventilator-associated pneumonia (VAP). The median number of previous pneumonia cases per patient was two (IQR: 1).

Samples collected were primarily sputum (96.39%, n = 80), with bronchoalveolar lavage fluid (BALF) comprising three samples (3.61%). The C-reactive protein (CRP) levels at diagnosis had a median value of 89 (IQR: 106.5), while at discharge, it was 80.65 (IQR: 111.8). Regarding ventilation support, the median duration of invasive mechanical ventilation was 3 days (IQR: 18), and oxygen therapy lasted a median of 5.5 days (IQR: 19) among the participants. Findings about outcomes represented that almost half of the patients (51.85%, n = 42) survived, whereas 39 (48.15%) did not survive.

### 2.3. Microbiological Data

**Figure 1:**
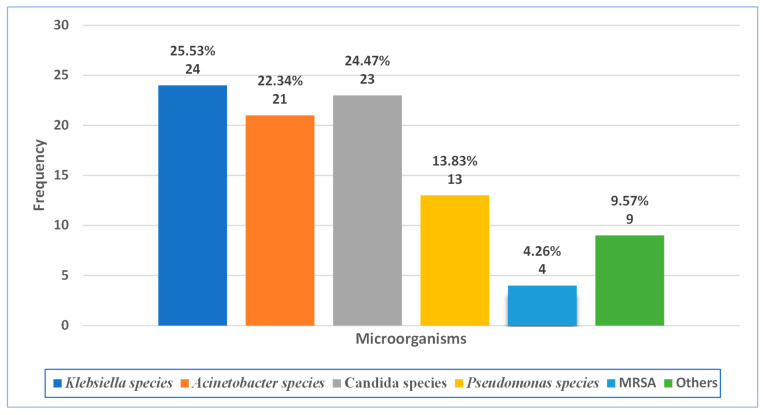
Microbial distribution. This figure shows the frequency and percentage of detected microorganisms using culture (N = 94, total number of positive culture results).

The analysis of the distribution of different microorganisms (out of 94 positive culture results) is represented in a histogram in Figure 1. The results reveal that *Klebsiella* species, which include *Klebsiella pneumoniae*, are the most prevalent, accounting for 24 results (25.53%). *Candida* species follows with 23 (24.47%), while *Candida albicans* accounts for most of the findings (16/23). *Acinetobacter* species, including *Acinetobacter baumanii*, were responsible for 21 results (22.34%)*. Pseudomonas* species fell slightly lower, constituting 13 positive results (13.83%), including *pseudomonas aeruginosa*, which represented most of the species’ results (12/13). MRSA is relatively less frequent and presented only in four cases (4.26%). The “Others” category, which includes uncommon microorganisms causing HAP such as *Burkholderia cepacian***,** *Citrobacter freundii***,** and *Sphingomonas paucimobilis*, collectively represents a moderate proportion, with nine cases (9.57%).

### 2.4. Antimicrobial Use

Regarding our analysis of participants’ data about the use of various antimicrobial agents across different stages of HAP treatment, including empiric (241 selections), initial (65 selections), and next-line therapy (51 selections), the findings are listed in Table 3. Regarding carbapenem, meropenem was the most frequently selected empirically (16.60%) but decreased in use during the initial (7/65, 10.77%) and next (3/51, 5.88%) stages. Imipenem–cilastatin was used in 10.79% of empirical stages, 4.62% of initial stages, and 3.92% of next stages. Notably, piperacillin–tazobactam, which is a penicillin antibiotic, was used empirically in 26 cases (10.79%), with limited continued use in subsequent stages. Cephalosporins and macrolides were not frequently used in the different stages of treatment. Vancomycin showed the highest empirical use among all antimicrobial agents (49/241, 20.33%), with (7/65, 10.77%) in the initial and (4/51, 7.84%) in the next stages. Quinolones, represented by levofloxacin, had 11.62% empiric use, which decreased to 9.80% in next-line therapy. Two aminoglycoside agents, amikacin and gentamicin, were used across empirical therapy, with prevalence values of 5.81% and 4.56%, respectively. Among the antifungals, anidulafungin was notably used in 10 out of 241 empirical selections (4.15%), and it was also used in other stages. Across the other antimicrobial options, colistin demonstrated significant growth (from 5.39% in the empiric stage to 21.54% in the initial stage).

### 2.5. Bacterial Sensitivity Toward Antibiotics

Data about sensitivity of most common causative bacteria culture results toward antibiotics across the participants were analyzed, and the results are provided in Table 4. Regarding the findings, *Acinetobacter baumannii* bacteria showed 100% resistance to most antibiotics, including imipenem, meropenem, and piperacillin–tazobactam. Colistin was the most effective, with 92.31% sensitivity. Ceftazidime and ciprofloxacin showed lower sensitivities of 7.14% and 7.69%, respectively. Results regarding *Klebsiella pneumoniae* showed that meropenem, piperacillin–tazobactam, and most of cephalosporins achieved 100% resistance. Additionally, amoxicillin–clavulanic acid and ceftolozane–sulbactam had 100% resistance toward detected *Klebsiella pneumoniae*. Ciprofloxacin showed a low sensitivity at 5.56%, while amikacin had 61.11% intermediate sensitivity. Colistin exhibited a 44.44% sensitivity rate, with 55.56% resistance. Tigecycline was also highly effective toward *Klebsiella pneumoniae*, with 85.71% sensitivity. Regarding *Pseudomonas aeruginosa*, colistin showed the highest sensitivity at 100%, followed by imipenem at 90%. Ceftazidime showed the lowest sensitivity toward *Pseudomonas aeruginosa* at 30% frequency. The analysis showed that Oxacillin showed 100% resistance toward MRSA, while vancomycin demonstrated 100% sensitivity. Clindamycin and levofloxacin each had 50% sensitivity toward MRSA, highlighting partial effectiveness.

### 2.6. Culture and qPCR in Microbial Detection

The data presented in Table 5 shows a detailed comparison of detection for microorganisms identified through two diagnostic methods: culture and qPCR conducted using Chi-square test. *Acinetobacter baumanii* exhibited the highest frequency across both methods, being detected in 19.28% (16/83) of culture cases and 62.20% (51/82) of qPCR cases, with a *p*-value of 0.02, also showing a statistically significant difference between the two methods. *Klebsiella pneumoniae* also demonstrated significant presence, with detection rates of 22.89% (19/83) in culture and 45.12% (37/82) in qPCR. Also, the *p*-value was highly significant (*p*-value < 0.001). *Pseudomonas aeruginosa* showed a culture-positive rate of 14.46% and a qPCR-positive rate of 13.42%. Despite these similar values, the *p*-value was <0.001, suggesting a strongly significant result. In contrast, *Enterobacteriaceae* was dominant only in qPCR at 46.34% (38/82). Also, *Escherichia coli* was only detected with qPCR in 12.16%, while *Proteus* species and *Klebsiella oxytoca* were among the least frequently detected in culture and qPCR. Surprisingly, the *p*-value showed a highly significant difference (*p*-value < 0.001). Additionally, *Enterobacter cloacae* was moderately prevalent with qPCR compared to culture, and the *p*-value was again statistically significant (*p*-value = 0.004). On the other hand, *Candida* species were prominent in culture, with prevalence of 18.07% (15/83). In addition, other *Candida* species, such as *Candida tropicalis* and *Candida krusei*, only were detected in culture with a notably low percentage.

Additionally, the relationship between the total number of positive microbial culture detections and real-time qPCR-positive microbial detections per patient using Pearson Correlation is shown in Table 6. The results showed that real-time qPCR can detect a higher number of microorganisms across the participants. The test result of 0.287 gives a weak to medium correlation between the two methods. This may predict a presence of difference between two methods in detection. The *p*-value was statistically significant.

### 2.7. Antibiotic Resistance Gene Detection

#### 2.7.1. Antibiotic Resistance Gene Prevalence

The prevalence of various resistance genes in the study samples with positive microorganisms, established using qPCR, is summarized in Table 7. Among carbapenem resistance genes, the *ndm* gene showed the highest prevalence, being found in 39 samples (60%), followed by the *oxa-23*, *oxa-58*, and *oxa-48* genes, which were detected in 38 out of 65 samples, with 58.46% prevalence. The *oxa-51* gene was abundant in 22 samples (33.85%), while the *vim* gene was in 4 samples (6.15%), and the *imp* gene was present in only 2 samples (3.078%). Lastly, the *kpc* gene was not detected. In a subset of five samples with positive *staphylococcus aureus*, the vancomycin resistance gene *vanA*/*B* appeared only in one sample (20%). Related to the quinolone resistance gene, the *qnr* gene was found in 48.44% of the 64 samples. The *CTX-M* resistance gene of ESBL was detected in 40.63% of the 64 samples. The methicillin resistance genes *mecA*/*mecC* were present in half of the 64 samples.

#### 2.7.2. Culture and qPCR Antibiotic Resistance Detection

Table 8 provides a detailed analysis of resistance gene detection by qPCR and sensitivity culture results for various antibiotics, and analysis of the difference between them was performed using a Chi-square test. For imipenem, 76.60% of the isolates were resistant, and 23.40% were sensitive. Among the OXA resistance genes, the *oxa-48* gene had the highest detection rate among the resistant isolates, 88.89%, showing significant association (*p*-value = 0.004). The *oxa-23* and *oxa-58* genes had a high and equal prevalence among resistance samples but were without a statistically significant association *p*-value. The detection rate of overall OXA mix resistance genes was 97.22% among resistant cases toward imipenem, with a statistically significant *p*-value of 0.023. Regarding CRE resistance gene detection for imipenem, the *ndm* gene had the highest detection rate of 80.56%. In contrast, the *imp* gene was rarely detected, with a 2.78% rate and *p*-value = 0.855. The *kpc* resistance gene was not detected in all resistance isolates. The overall *CRE* mix was detected in 30 out of 36 resistant sensitivity results for imipenem. Overall, qPCR was able to detect the presence of at least one carbapenem resistance gene in all resistant isolates toward imipenem antibiotics, with a statistically significant association (*p*-value = 0.003).

Meropenem showed a higher resistance rate (83.78%). The overall *OXA* mix detection rate toward resistant results was 96.77%, with *oxa-48*, *oxa-58*, and *oxa-23* being highly prevalent among these resistant genes, although the association was not statistically significant. Among *CRE* mix genes, *vim* had a low prevalence rate among resistance cases (3.23%), and *imp* and *kpc* were not detected in all resistance cases toward meropenem. The CRE mix genes were detected in 80.65% of non-susceptible results of meropenem. The overall detection rate of carbapenem resistance genes in resistant meropenem isolates was 100% and statistically significant.

Ertapenem exhibited the highest resistance rate of 89.66%. Through resistance strains towards ertapenem, *OXA* mix resistance gene detection, representing the resistance results by culture, showed 100% frequency and a strong association *p*-value (*p*-value ≤ 0.001). Again, *oxa-48* was the most frequently detected oxa gene toward ertapenem (92.31%). Additionally, *CRE* mix and the overall carbapenem resistance gene detection rate in ertapenem-resistant strains were 96.15% and 100%, respectively, with a *p*-value of <0.001 for both. The *ndm* gene detection rate was the highest, with a statistically significant *p*-value of <0.001.

For fluoroquinolones, levofloxacin had a resistance prevalence of 87.5%, with *qnr* and *CTX-M* sharing a detection rate of 35.71% among resistant cases but having different association *p*-values of 0.696 and 0.308, respectively. On the other hand, ciprofloxacin had a higher detection rate between resistance isolates of *qnr* compared to *CTX-M*, albeit without statistically significant *p*-values for both.

Among β-lactams, resistance to ampicillin–sulbactam was highest (92.31%), but the *CTX-M* resistance gene was detected in around half of the sensitivity results, without a statistically significant association, followed by amoxicillin–clavulanic acid, with a resistance prevalence of 91.67% and a high *CTX-M* detection rate of 72.73%. Piperacillin–tazobactam and aztreonam had moderate *CTX-M* gene detection frequency between resistance strains, though the *p*-value was insignificant. Between cephalosporins, the ceftriaxone resistance frequency was 93.94%, showing a *CTX-M* detection rate of 61.29%. In contrast, ceftazidime–avibactam had the lowest resistance frequency but the highest *CTX-M* detection frequency among the resistance isolates (71.43%). Both ceftriaxone and ceftazidime–avibactam had a non-significant *p*-value, representing the association between *CTX-M* detection and resistance isolates.

Regarding aminoglycosides and the among culture results, gentamicin and amikacin had almost the same resistance rate. The *CTX-M* detection percentages through resistance cases toward gentamicin and amikacin were 60% and 50%, respectively. The association *p*-value for aminoglycoside was not statistically significant. Similarly, the trimethoprim–sulfamethoxazole *CTX-M* gene detection rate had an insignificant *p*-value (0.199).

#### 2.7.3. Mortality Risk

In our analysis, we sought to test the most prevalent resistance genes among the participants as predictors for mortality in HAP patients. The prediction variables were *oxa-23*, *oxa-48*, *oxa-51*, *qnr*, *mecA*/*mecC*, and *CTX-M* resistance genes. The results of the regression model test showed that none of these resistance genes had a significant dds ratio. This may be due to the limited sample size of the study.

## 3. Discussion

In our study, we used real-time PCR to detect the most common causative bacteria of hospital-acquired pneumonia. Furthermore, we selected resistance genes of bacteria toward antibiotics, detected using real-time PCR, because of their importance in influencing the improvement of pneumonia management. The selected resistance genes included the following:

- Carbapenem resistance genes: *CRE* genes (*KPC*, *NDM*, *VIM*, *IMP*) and OXA genes (*OXA-51*, *OXA-23*/*OXA-58*, *OXA-48*);
- Quinolone resistance gene: *qnr*;
- Methicillin resistance genes: *mecA* and *mecC*;
- ESBL resistance gene: *CTX-M*;
- Vancomycin resistance genes: *VanA*/*VanB*.

Our aim was to evaluate the performance of the qPCR assay for detecting causative bacteria of hospital-acquired pneumonia. Also, an important objective was to demonstrate the frequency of resistance genes among bacteria toward commonly prescribed antibiotics for pneumonia patients in The University of Jordan hospital and to determine if resistance gene detection by real-time qPCR is representative of the overall susceptibility results revealed by previous traditional methods. Based on these aims, we ran our qPCR procedure using lower respiratory tract and the resistance gene panels, and then, we compared the findings with culture results collected from medical profiles.

New molecular techniques in diagnosis of pneumonia have facilitated the proper initiation of antimicrobial therapy, identifying microorganisms including bacteria, viruses, and fungi, responsible for infection [9]. This contributes to better outcomes for pneumonia patients [10].

Several previous studies have found that real-time PCR is the gold standard for diagnosis of several infections such as COVID-19 because of its exceptional efficacy in detecting low amounts of multiple genetic material [11,12,13,14]. One such study on the use of real-time qPCR in detection of SARS-CoV-2 based on sputum samples revealed a sensitivity and specificity of 86% and 37%, respectively [15].

A major drawback of the PCR molecular analysis technique is that bacterial detection rate is affected in prior antibiotic use [16]. In a study of molecular technique using diagnosis of lower respiratory tract samples of CAP patients, the results showed that molecular testing greatly enhances pathogen detection, especially in patients exposed to antibiotics compared with culture [17]. Regarding this, the empiric antibiotic use in our study may have affected the detection of causative bacteria. However, in general, real-time PCR is still able to detect bacteria with a previous antibiotic administration at a higher rate compared with the conventional culture method.

In terms of detecting respiratory pathogens, especially Gram-Negative Bacteria (GNB), and identifying common patterns of antibiotic resistance, molecular assays exhibit good agreement with standard-of-care diagnostics and have better analytical sensitivity than cultural approaches [18]. Consistent with the findings of previous studies, the results of our study indicate significant differences between traditional culture and qPCR in terms of detection of important pathogens causing severe respiratory infections such as MDR-GNB (e.g., *Klebsiella* species, *Acinetobacter* species, and *Enterobacter* species). This finding aligns with the well-known importance of early use of empirical antibiotics, which suggests that correct microbial diagnosis can achieve this.

In addition, this study emphasizes that identification of causative pathogens by real-time PCR was more frequent than by conventional methods in many of the cases. This implies that as the complexity of the infection increases, so does the ability of qPCR to identify the multiple microorganisms that coexist and cause the infection. This will be considered in antimicrobial selection, as the infection is more complicated and may be associated with higher non-susceptibility toward different antibiotic agents.

Regarding emerging resistance toward antibiotics, bacterial pathogens have evolved many strategies to evade the bactericidal effects of antimicrobial agents [19]. In alignment with this, in cases where patients were initiated on antibiotics before sample collection, administration of this antibiotic may have triggered the emergence of new resistance genes. Phenotyping resistance does not necessarily correspond with the presence of resistance markers. Reverse transcriptase qPCR, which measures RNA, could possibly solve this issue [20].

The choice of empirical therapy in treatment of HAP is challenging because it depends on the risk factors for MDR pathogens, which increases mortality risk [3]. In a large, multicenter study in Jordan about MDR *Acinetobacter baumannii*, it was found that 76.8% of the isolates were not susceptible to most of the antibiotics [21]. In another study of resistance to aminoglycoside and quinolone drugs across *Klebsiella pneumoniae* in northern Jordan, the rates of resistance to aminoglycosides and quinolones were 65.0% and 61.7%, respectively [22]. In our analysis, regarding the susceptibility results derived from culture toward *Acinetobacter baumannii*, the resistance rate was 100% for most antibiotic options, and colistin sensitivity was around 92%. In addition, among *Klebsiella pneumonia* isolates, most antibiotics were resistant, and tigecycline had the highest sensitivity. Considering this, selection of tigecycline as the initial antibiotic in pneumonia patients infected with *Klebsiella pneumonia* bacteria, who do not have contraindications toward tigecycline use, may have a good impact on the treatment outcome.

In a study about prevalence of MRSA in southern and eastern Mediterranean countries, for most of the countries facing a rise in MRSA infections, the percentage was more than 50% in Jordan [23]. Among antibiotic the resistance genes encoding ESBLs, the *CTM-X* resistance gene is extremely widespread across nosocomial settings [24]. Carbapenem resistance genes have become very prevalent and hinder the effectiveness of carbapenem use nowadays. OXA-48-like β-lactamases are abundant worldwide [25]. *Enterobacteriaceae* and *Klebsiella pneumoniae* isolates carrying the *NDM* resistance gene are also commonly found in many countries [26].

In a previous study about multiplex real-time qPCR detection of bacterial respiratory pathogens and their microbial resistance genes, it was concluded that real-time qPCR was sensitive and specific [27]. Another study regarding the use of multiplex real-time PCR in detection of tigecycline resistance genes revealed that real-time PCR has the ability to detect genes not only present in bacteria but also in environmental samples [28]. Use of molecular techniques instead of conventional culture in pneumonia diagnosis is still not applicable. This is due to the limited ability of molecular techniques to detect specific pathogens and resistance markers involved in the designed assays [18]. As shown in our study, the method does not offer general detection of many possible microbial causes and non-susceptibility genomes in pneumonia.

Elaborating on previous studies, our work investigated whether real-time qPCR could be a rapid and effective in detecting emerging resistance genes toward many effective antibiotic classes. The frequencies of detected carbapenem, quinolone, ESBL, and methicillin resistance genes, derived from using real-time qPCR, were around 50% among the infected patients. Regarding the comparison between qPCR resistance gene detection and traditional culture susceptibility patterns, the results showed that presence of any carbapenem resistance genes, OXA genes, and *CRE* genes could be representative of the overall susceptibility phenotype. Additionally, the results found that *oxa-48* and *ndm* genes were mostly abundant among resistance isolates toward carbapenem. These two genes may be more associated with the carbapenem resistance phenotype. Our findings also demonstrated that the ESBL emerging resistance gene *CTX-M* were common among resistance isolates toward different classes of antibiotics, especially β-lactam. Meanwhile, the quinolone resistance gene *qnr* was frequently prevalent among ciprofloxacin-resistant isolates and, to a lesser extent in, levofloxacin-resistant isolates.

Early response to antibiotic therapy can be observed by using effective and rapid diagnostic methods, which offer important insights into treatment success. A notable early initiation of antibiotic correctness could change pneumonia patient outcomes. Additionally, to improve the outcome after antibiotic therapy initiation, microbial susceptibility toward antibiotics is crucial. This combination of proper microbial diagnosis and related sensitivity toward antimicrobial choices, evaluated using a rapid and valid technique such as real-time qPCR, may be a cornerstone in increasing the survival rate of pneumonia patients at The University of Jordan hospital.

The discussion of our study closely matches the elements that have been found to influence responses to antibiotics and how well pneumonia is managed overall. Notably, there is not much published research about real-time PCR use in pneumonia management in Jordan. The purpose of our study is to fill this gap by providing information on how using real-time qPCR is influencing treatment success in patients who have acquired pneumonia after hospitalization and/or ventilation.

This study has some limitations that should be acknowledged. First, it was conducted at a single center, which may limit the generalizability of the results to other healthcare settings with different patient populations or clinical practices. Second, the relatively small sample size reduces the statistical power of the study and may have limited the ability to detect less pronounced associations or differences between groups. Third, the study duration was relatively short (six months), which means it may not fully capture seasonal variations or long-term trends in pathogen prevalence and resistance patterns. Additionally, the possibility of *Candida* isolation representing colonization or contamination rather than real infection cannot be excluded, potentially influencing the interpretation of the microbiological findings.

### Future Work

For future work, the following are the main suggestions:

1. Optimization of new kits that include a larger spectrum of target pathogens to be detected using real-time PCR and involving other, less common causative pathogens such as fungi (e.g., *Candida albicans*, *Candida tropicalis*);
2. Validate the efficacy, selectivity, and sensitivity of real-time PCR in detecting lower respiratory tract microorganisms in a larger-sized sample and via a multicenter study of HAP patients;
3. Consider other important emerging resistance genes in the University of Jordan hospital toward effective antibiotics such as colistin;
4. Study the most prevalent resistance genes in The University of Jordan hospital as mortality risk predictors.

## 4. Materials and Methods

### 4.1. Study Design

This prospective observational study used a molecular biology approach for microbial diagnosis and detection of antibiotic-resistance genes in bacterial strains isolated from hospitalized HAP patients at The University of Jordan Hospital. Clinical data from medical files were used to collect and analyze demographic and clinical information, including clinical outcomes.

### 4.2. Study Sample Selection

Lower respiratory tract samples from the hospital were collected from HAP patients (including VAP patients) between May 2024 and October 2024. Inclusion and exclusion criteria are summarized in Table 9.

#### 4.2.1. Sample Types

**Figure 2:**
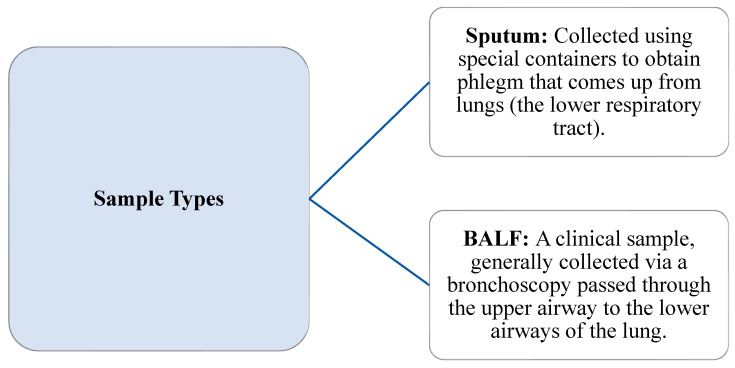
Study sample types.

For this study, the specimens included were sputum and bronchoalveolar lavage fluid (BALF) samples. Figure 2 provides an overview of the sample types.

#### 4.2.2. Handling and Storage

Samples were promptly transported to the laboratory and stored at −80 °C after being mixed with DNA/RNA Shield (ZYMO Research, Irvine, CA, USA) (1:1 volumes) to preserve the integrity of the genetic material until processing.

### 4.3. Clinical Data and Outcomes

Patient medical records from The University of Jordan Hospital were used to extract relevant data about gender, age, and comorbidities. In addition, clinical data about pneumonia classification (HAP or VAP), risk factors for HAP and or VAP, location of hospitalization, symptoms onset, vital signs, lab results, and clinical findings were considered. These data, together with microbiological findings, were collected to make a clinical judgment about the antibiotic used in treatment. Outcomes of interest were improvement status and time to clinical improvement. Clinical data and outcomes were collected using a case report form, overviewed in Table 10.

The bacteria were identified through culture using the vitek 2 compact automated system, an automated system developed for microbial identification and antimicrobial susceptibility testing (AST) in both clinical and industrial lab settings.

### 4.4. DNA Extraction

**Figure 3:**
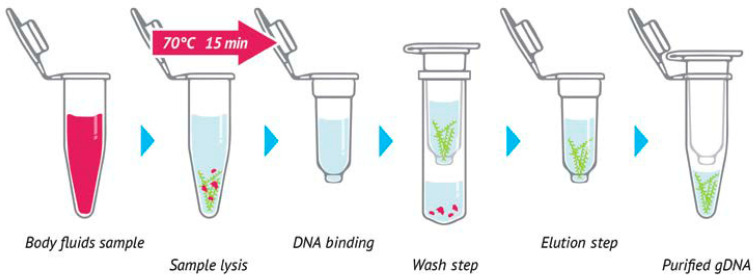
DNA extraction steps.

DNA extraction was performed using the NZY Bacterial & Viral RNA/DNA Isolation Kit (NZYtech Lda, Lisboa, Portugal) according to the manufacturer’s instructions (Figure 3). Samples of 200 µL were processed according to the kit protocol.

#### 4.4.1. Materials and Equipment

1. Proteinase K solution (Qiagen, Hilden, German)

This solution is designed to be used for protein degradation. It is used during sample preparation for viscous samples to ease their lysis.

2. Lysis buffer

NBVL buffer is a lysis solution used during the cell lysis step. It contains guanidine thiocyanate, which breaks down the cells and inactivates cellular RNAse and DNAse to prevent nucleic acid degradation.

3. Ethanol

Absolute ethanol (96–100%) is added to the samples to yield a homogenous solution and to ensure efficient binding of nucleic acids to the silica membrane column.

4. Washing buffer

NBV and NBVW buffers are used throughout the washing step. Washing buffers generally contain alcohols, which remove proteins, salts, and other contaminants from the sample or the upstream binding buffers.

5. RNase-free water

RNase-free water is used in the elution process, in addition to spinning, to remove the DNA from the filter, thus pulling the DNA into the collection tube.

6. NZYspin Bacterial/Viral column

Columns with silica membrane as a solid matrix packed in a column that selectively binds to DNA during the binding process and separates DNA from other remnants during the washing process.

7. Collection tubes (2 mL)

These tubes are used to discard the flow-through during the binding and washing steps, also being used to collect the final eluted DNA after elution.

8. ml Microcentrifuge tubes (Labselect, Beijing, China);
9. Micropipettes (Acura 825, Socorex, Ecublens, Switzerland);
10. Sterile disposable tips (Tarsons, Kolkata, India);
11. Centrifuge (MPW-260R, MPW, Warsaw, Poland);
12. Vortex mixer (KMC-1300V, Vision Scientific Co., Ltd., Daejeon, Republic of Korea);
13. Thermostatic Water Bath (Gemmy Industrial Corp., Taiwan, China).

#### 4.4.2. Sample Preparation

Excessively viscous samples were pre-treated with 15 μL of proteinase K and mixed vigorously via vortex. Then, they were incubated for 2–3 min at room temperature to facilitate cell lysis.

#### 4.4.3. Cell Lysis

In brief, 200 μL of each sample was combined with 350 μL of NBVL buffer, then vigorously vortexed and incubated for 10 min at room temperature (15–25 °C). Difficult-to-lyse samples were incubated for 15–20 min in a water bath at 65 °C.

#### 4.4.4. DNA Binding

First, 350 μL of absolute ethanol was added into samples and immediately mixed by vortex. Next, up to 700 μL of the ethanol-treated sample was transferred into an NZYSpin Bacteria/Viral column set in a 2 mL collection tube. Then, centrifugation took place at 800× *g* for 1 min. The flow-through was discarded and returned to the same collection tube.

#### 4.4.5. Washing

Briefly, 200 μL of NBV buffer was added to the column and centrifuged for 1 min at 8000× *g*. The flow-through was discarded and returned to the column via the same collection tube. Then, 600 μL of NBVW buffer was added to the column and centrifuged for 1 min at 8000× *g*. Also, the flow-through was discarded and returned to the column via the collection tube.

#### 4.4.6. Dry Silica Membrane

The column was centrifuged for an additional 2 min at 8000× *g* to ensure the column membrane was completely dry. Any flow-through collected was discarded.

#### 4.4.7. DNA Elution

The NZYSpin Bacterial/Viral column was placed in a clean RNase-free microcentrifuge tube, and 50–100 μL of pre-warmed (60–70 °C) RNase-free water was added directly onto the column membrane. The column was incubated with water at room temperature for 2–5 min. To collect the eluted DNA, we performed centrifugation for 1 min at 8000× *g*. Lastly, the eluted RNA/DNA was stored at −80 °C.

### 4.5. Real-Time PCR for Lower Respiratory Bacteria Detection

Real-time PCR was performed using the Lower Respiratory Bacteria qPCR Panel (Bioeksen AR GE Teknolojileri A.Ş, İstanbul, Türkiye) according to the manufacturer’s instructions to detect the most common microorganism in HAP.

#### 4.5.1. Materials and Equipment

1. Negative Control: A no-template control (NTC) allowing for contamination control during qPCR;
2. Positive Control (PC): We used a PC containing an artificial DNA sequence that corresponds to lower respiratory bacteria oligonucleotide (LRB Oligo) mix targets and the primer set that detects it, working as a reagent stability control (six PCs: PC-LRB 1/PC-LRB 2/PC-LRB 3/PC-LRB 4/PC-LRB 5/PC-LRB 6);
3. qPCR Mix: We used an optimized ready-to-use mix for the qPCR assay. The mixture contains all components necessary for PCR, including a green intercalating dye, dNTPs, stabilizers, and enhancers;
4. LRB Oligo Mix: This mix contained specific nucleic acid amplification and detection targets. LRB Oligo Mixes and their specific nucleic acid detection targets are summarized in Table 11;
5. Micropipettes (Biosan, Riga, Lativa);
6. Compatible filtered pipette tips (nuclease-free): Suitable for transferring 1–10, 10–100, and 100–1000 μL of liquid (Stent, Beijing, China);
7. Eppendorf tubes (Extra gene, Taiwan, China);
8. PCR plate (Genetics company, Shenzhen, China);
9. PCR plate seal (Genetics company, China);
10. Vortex for PCR plates (CVP-2, Biosan, Lativa);
11. Centrifuge (Combi-Spin, Biosan, Lativa);
12. Real-Time PCR Instrument (Quant Gene 9600, Bioer, Tokyo, Japan).

#### 4.5.2. Procedure

1. The PCR kit was taken out of the −20 °C freezer.
2. LRB Oligo Mixes and 2X qPCR Mix were spun via mini spin.
3. (Sample Count + 3) × 5 µL of LRB Oligo Mix 1 was pipetted into an empty Eppendorf tube.
4. (Sample Count + 3) × 10 µL of 2X qPCR Mix was added into the Eppendorf tube prepared in step 2.
5. The master mix was spun, using mini spin, to become homogenous.
6. Steps 3, 4, and 5 were repeated for all master mixes (6 master mixes in total).
7. Next, 15 μL of each master mix was pipetted into the relative PCR wells.
8. Then, 5 μL of each extracted sample was mixed by pipetting up and down into the relative PCR wells.
9. Then, 5 μL of NTC was mixed by pipetting up and down into negative control PCR wells.
10. Next, 5 μL of PC-LRB 1 was mixed by pipetting up and down into the first well of the PC plate, repeated for all PCs.
11. The PCR plate was sealed firmly and spun via vortex for each PCR plate for 30 s.
12. The qPCR device was programed according to the manufacturer’s qPCR program protocol. qPCR program protocol details are summarized in Table 12.
13. The lid of the instrument was opened to place the PCR plate and then closed so that the instrument could be used for 40 min.

### 4.6. Real-Time PCR for Resistance Gene Detection

Real-time PCR was performed using the Carbapenem Resistance qPCR Kit, the Vancomycin Resistance qPCR Kit, and the Urinary Tract Antibiotic Resistance qPCR Panel (Bioeksen AR GE Teknolojileri A.Ş, İstanbul, Türkiye), according to the manufacturer’s instructions. This procedure was performed to detect carbapenem, quinolone, extended-spectrum β-lactamases (ESBL), and vancomycin resistance genes.

#### 4.6.1. Materials and Equipment

1. Urinary Tract Antibiotic Resistance qPCR Panel kit (qPCR Mix, Urinary tract antibiotic resistance (UTABR) Oligo Mix 2/3, PC-UTABR 2/3, NTC);
2. Carbapenem Resistance qPCR Kit (qPCR Mix, Carbapenem resistance *Enterobacteriaceae* (*CRE*) Oligo Mix, OXA Oligo Mix, PC-CRE, PC-OXA, NTC).

We used the Vancomycin Resistance qPCR Kit (qPCR Mix, Vancomycin-resistance *Enterococci* (VRE) Oligo Mix, PC-VRE, NTC). Oligo Mixes for each kit and their specific resistance gene detection targets are summarized in Table 13.

3. Micropipettes (Biosan, Lativa);
4. Compatible filtered pipette tips (nuclease-free) (Stent, China);
5. Eppendorf tubes (Extra gene, Taichung, Taiwan);
6. PCR plate (Genetics company, China);
7. PCR plate seal (Genetics company, China);
8. Vortex for PCR plates (CVP-2, Biosan, Lativa);
9. Centrifuge (Combi-Spin, Biosan, Lativa);
10. Real-time PCR instrument (Quant Gene 9600, Bioer, Japan).

#### 4.6.2. Procedure

1. The PCR kit was taken out of the −20 °C freezer.
2. UTABR Oligo Mixes, *CRE* Oligo Mixes, and VRE Oligo Mix with 2X qPCR Mix for each kit were spun using mini spin.
3. (Sample Count + 3) × 5 µL of UTABR Oligo Mix 2, CRE Oligo Mix, and VRE Oligo Mix was pipetted into an empty Eppendorf tube for each one.
4. (Sample Count + 3) × 10 µL of 2X qPCR Mix for each kit was added into the related Eppendorf tube prepared in step 2.
5. The master mix was spun, using mini spin, to become homogenous.
6. Steps 3, 4, and 5 were repeated for all master mixes (5 master mixes in total).
7. Then, 15 μL of each master mix was pipetted into the relative PCR wells.
8. Next, 5 μL of each extracted sample was mixed by pipetting up and down into the relative PCR wells.
9. Then, 5 μL of each NTC was mixed by pipetting up and down into related Negative Control PCR wells.
10. Next, 5 μL of PC- UTABR 2/3, PC-*CRE*/OXA, and PC-VRE were mixed by pipetting up and down into the first relative well of the PC plate, and this was repeated for all PCs.
11. The PCR plate was sealed firmly and spun via vortex for all PCR plates for 30 s.
12. The qPCR device was programed according to the same manufacturer’s qPCR program protocol used during the lower respiratory bacteria detection procedure.
13. The lid of the instrument was opened to place the PCR plate and then closed to use the instrument for 40 min.

### 4.7. Statistical Analysis

Data were analyzed using Jeffreys’s Amazing Statistics Program (JASP) v.0.19.1.0 (Amsterdam, The Netherlands) and Statistical Package for the Social Sciences (SPSS) statistics v.24 (IBM Corporation, New York, NY, USA). The Shapiro–Wilk test for normality was applied. A *p*-value of less than 0.05 was considered statistically significant.

A comparison of detection rates for microorganisms and resistance genes between the traditional culture and qPCR was conducted with the Pearson Chi-Square test.

A Pearson correlation test was run to determine whether there was a relationship between the total number of positive culture results and real-time qPCR-positive results per patient.

Lastly, mortality risk prediction of prevalent resistance genes between the participants was performed using a logistic regression analysis (odds ratio).

## 5. Conclusions

To manage hospital-acquired pneumonia effectively, it is essential to diagnose the causative microorganisms and determine the susceptibility of resulting bacteria toward different antibiotics. This study showed that the real-time PCR technique, combined with data on the epidemiology of antibiotic resistance genes, could be used to create a useful tool with attributes to improve HAP management.

Our investigation focused on determining the most common bacteria that could cause hospital-associated pneumonia and identifying resistance genes toward effective antibiotics in the treatment of HAP in The University of Jordan hospital. Considering that this study has shown how effective qPCR is at detecting bacteria and related resistance genes, it also has certain limitations, namely its limited sample size and the fact that it was not designed as a longitudinal study. In addition, a more comprehensive multicenter study is required to study real-time PCR use in the management of HAP in Jordan.

## Figures and Tables

**Table 1:** Demographics and clinical features of the study participants (N = 83).

Variables	Mdn (IQR)	
Age	63 (36.5)	
Variables	N	%
Gender		
Male	51	61.45%
Female	32	38.55%
Comorbidities		
Cardiovascular diseases	48	57.83%
Diabetes Mellitus	32	38.55%
Malignancy	20	24.10%
CKD	19	22.89%
Another Lung disease *	19	22.89%
Hepatic Impairment	6	7.23%
Others	15	18.07%
Risk Factors for HAP &/or VAP		
Stroke or other neurologic disorders, seizures	30	36.14%
Use of antibiotics in the last 3 months	28	33.73%
Intubation and mechanical ventilation	27	32.53%
Tube feeding	26	31.33%
Supine positioning of the patient	24	28.92%
Hyperglycemia	7	8.43%
Aspiration	7	8.43%
Others	5	6.02%
Initial Findings		
Chest radiography (segmental infiltrate)	60	72.29%
Tachycardia (HR > 100 beats/min)	25	30.12%
Altered breath sounds or localized rales	22	26.51%
Cough and sputum production	21	25.30%
SOB	20	24.10%
Tachypnoea	18	21.69%
Hypotension (BP < 90 mmHg)	12	14.46%
Vomiting	6	7.23%
Loss of appetite	6	7.23%
Others	9	10.84%

* other lung disease includes cystic fibrosis, bronchiectasis, lung fibrosis, and respiratory failure.

**Table 2:** Clinical data and outcome for the study participants (N = 83).

Variables	Mdn (IQR)	N (%) *
Number of Previous Hospitalization	5 (6)	
Hospitalization Location		
Ward		31 (37.35%)
ICU		52 (62.65%)
Hospital Length of stay (LOS)	25 (28)	
ICU LOS	19.5 (22.75)	
Pneumonia Classification		
HAP (excluding VAP)		60 (72.29%)
VAP		23 (27.71%)
Number of Previous Pneumonia	2 (1)	
Sample Type		
Sputum		80 (96.39%)
BALF		3 (3.61%)
C-Reactive Protein (CRP)		
At diagnosis	89 (106.5)	
At discharge	80.65 (111.8)	
Total duration (days) of ventilation		
Invasive mechanical ventilation	3 (18)	
O_2_ therapy	5.5 (19)	
Outcome		
Survived		42 (51.85%)
Not Survived		39 (48.15%)

* represents valid percent.

**Table 3:** Antimicrobial selection strategy for the participants.

Antibiotic	Antibiotic Selection Strategy (N %)		
	Empiric * (*n* = 241)	Initial ** (*n* = 65)	Next *** (*n* = 51)
Carbapenem			
Imipenem–cilastatin	26 (10.79%)	3 (4.62%)	2 (3.92%)
Meropenem	40 (16.60%)	7 (10.77%)	3 (5.88%)
Ertapenem	1 (0.41%)	-	1 (1.96%)
Penicillin			
Piperacillin–tazobactam	26 (10.79%)	-	2 (3.92%)
Cephalosporin			
Ceftriaxone	4 (1.66%)	-	-
Ceftazidime–avibactam	2 (0.83%)	3 (4.62%)	-
Others	1 (0.41%)	1 (1.54%)	2 (3.92%)
Macrolide			
Azithromycin	3 (1.24%)	-	-
Erythromycin	-	1 (1.54%)	-
Glycopeptide			
Vancomycin	49 (20.33%)	7 (10.77%)	4 (7.84%)
Teicoplanin	-	-	1 (1.96%)
Quinolone			
Levofloxacin	28 (11.62%)	-	5 (9.80%)
Aminoglycoside			
Amikacin	14 (5.81%)	6 (9.23%)	3 (5.88%)
Gentamicin	11 (4.56%)	5 (7.69%)	3 (5.88%)
Antifungal			
Anidulafungin	10 (4.15%)	3 (4.62%)	5 (9.80%)
Voriconazole	1 (0.41%)	1 (1.54%)	3 (3.614%)
Others	4 (1.66%)	-	2 (3.92%)
Other antimicrobial agents			
Colistin	13 (5.39%)	14 (21.54%)	7 (13.73%)
Tigecycline	2 (0.83%)	6 (9.23%)	5 (9.80%)
Trimethoprim–sulfamethoxazole	2 (0.83%)	3 (4.62%)	2 (3.92%)
Others	4 (1.66%)	5 (7.69%)	1 (1.96%)

* empiric therapy involves antibiotics selected in the absence of definitive pathogen identification. ** initial therapy involves a targeted antibiotic selected after definitive pathogen and susceptibility identification. *** next represents next-line antibiotics used when initial therapy fails to achieve clinical improvement.

**Table 4:** Antibiotics’ sensitivity toward bacteria using culture.

Bacteria (N) *	Sensitivity N (%) **		
	Sensitive	Intermediate	Resistance
*Acinetobacter Baumannii* (16)	Amikacin		
	-	-	15 (100%)
	Aztreonam		
	-	-	5 (100%)
	Cefazolin		
	-	-	5 (100%)
	Cefepime		
	-	1 (6.25%)	15 (93.75%)
	Cefotaxime		
	-	1 (7.14%)	13 (92.86%)
	Cefoxitin		
	-	-	7 (100%)
	Ceftazidime		
	1 (7.14%)	-	13 (92.86%)
	Ceftriaxone		
	-	-	14 (100%)
	Ciprofloxacin		
	1 (7.69%)	-	12 (92.31%)
	Colistin		
	12 (92.31%)	-	1 (7.69%)
	Doxycycline		
	2 (28.57%)	-	5 (71.43%)
	Ertapenem		
	-	-	9 (100%)
	Gentamicin		
	2 (15.39%)	-	11 (84.62%)
	Imipenem		
	-	-	16 (100%)
	Levofloxacin		
	-	-	7 (100%)
	Meropenem		
	-	-	12 (100%)
	Minocycline		
	-	1 (14.29%)	6 (85.71%)
	Piperacillin–tazobactam		
	-	-	15 (100%)
	Tetracycline		
	-	-	7 (100%)
	Tobramycin		
	-	-	6 (100%)
	Trimethoprim–sulfamethoxazole		
	1 (20%)	-	4 (80%)
*Klebsiella pneumoniae* (19)	Amikacin		
	2 (11.11%)	11 (61.11%)	5 (27.78%)
	Amoxicillin–clavulanic acid		
	-	-	11 (100%)
	Cefazolin		
	-	-	11 (100%)
	Cefepime		
	1 (5.56%)	-	17 (94.44%)
	Cefotaxime		
	1 (9.09%)	-	10 (90.91%)
	Cefoxitin		
	-	-	6 (100%)
	Ceftazidime		
	1 (5.88%)	-	16 (94.12%)
	Ceftalozane–sulbactam		
	-	-	5 (100%)
	Ceftriaxone		
	-	-	16 (100%)
	Cefuroxime		
	-	-	8 (100%)
	Ciprofloxacin		
	1 (5.56%)	-	17 (94.44%)
	Colistin		
	4 (44.44%)	-	5 (55.56%)
	Ertapenem		
	1 (5.88%)	-	16 (94.12%)
	Gentamicin		
	2 (11.11%)	1 (5.56%)	15 (83.33%)
	Imipenem		
	1 (5.56%)	-	17 (94.44%)
	Meropenem		
	-	-	14 (100%)
	Piperacillin–tazobactam		
	-	-	15 (100%)
	Tigecycline		
	6 (85.71%)	-	1 (14.29%)
	Trimethoprim–sulfamethoxazole		
	10 (83.33%)	-	2 (16.67%)
*Pseudomonas aeruginosa* (12)	Amikacin		
	4 (44.44%)	-	5 (55.56%)
	Aztreonam		
	3 (37.5%)	-	5 (62.5%)
	Cefepime		
	4 (36.36%)	-	7 (63.64%)
	Ceftazidime		
	3 (30%)	-	7 (70%)
	Ciprofloxacin		
	4 (40%)	-	6 (60%)
	Colistin		
	3 (100%)	-	-
	Gentamicin		
	5 (62.5%)	-	3 (37.5%)
	Imipenem		
	9 (90%)	-	1 (10%)
	Meropenem		
	5 (50%)	-	5 (50%)
	Piperacillin–tazobactam		
	7 (63.64%)	1 (9.09%)	3 (27.27%)
MRSA (4)	Clindamycin		
	2 (50%)	-	2 (50%)
	Erythromycin		
	1 (25%)	-	3 (75%)
	Gentamicin		
	3 (75%)	1 (25%)	-
	Levofloxacin		
	2 (50%)	-	2 (50%)
	Oxacillin		
	-	-	4 (100%)
	Vancomycin		
	4 (100%)	-	-

* represents number of cases with detected bacteria using culture. ** represents valid percentages.

**Table 5:** Difference between culture- and qPCR-positive microbial detection.

Microorganism	Culture		qPCR		*p*-Value (Pearson Chi-Square Test)
	N	%	N	%	
*Proteus* species	1/83	1.21%	2/82	2.44%	<0.001
*Streptococcus pneumoniae*	-	-	4/82	4.88%	-
*Streptococcus pyogen*	1/83	1.21%	1/82	1.22%	-
*Streptococcus agalactiae*	-	-	1/82	1.22%	-
*Mycoplasma pneumoniae*	-	-	-	-	-
*Haemophilus influenzae*	-	-	4/82	4.88%	-
*Moraxella Catarrhalis*	-	-	1/82	1.22%	-
*Klebsiella aerogenes*	-	-	-	-	-
*Klebsiella oxytoca*	1/83	1.21%	4/82	4.88%	<0.001
*Enterobacteriaceae*	-	-	39/82	47.56%	-
*Escherichia coli*	-	-	10/82	12.16%	-
*Enterobacter cloacae*	1/83	1.21%	9/82	10.98%	0.004
*Serratia marcescens*	-	-	5/82	6.10%	-
*Legionella pneumophila*	-	-	-	-	-
*Pseudomonas aeruginosa*	12/83	14.46%	11/82	13.42%	<0.001
*Acinetobacter baumannii*	16/83	19.28%	51/82	62.20%	0.020
*Klebsiella pneumoniae*	19/83	22.89%	37/82	45.12%	<0.001
*Staphylococcus aureus*	-	-	5/82	6.10%	-
*Pandorea* species	1/83	1.21%	-	-	-
*Burkholderia cepacia*	3/83	3.61%	-	-	-
*Sphingomonas paucimobilis*	1/83	1.21%	-	-	-
Gram-negative-*Staphylococcus* species	1/83	1.21%	-	-	-
*Acinetobacter* species	5/83	6.02%	-	-	-
*Klebsiella* species	4/83	4.82%	-	-	-
*Pseudomonas* species	1/83	1.21%	-	-	-
MRSA	4/83	4.82%	-	-	-
*Candida albicans*	15/83	18.07%	-	-	-
*Candida tropicalis*	2/83	2.41%	-	-	-
*Candida krusei*	1/83	1.22%	-	-	-
*Candida glabrata*	3/83	3.61%	-	-	-
*Candida* species	1/83	1.21%	-	-	-

**Table 6:** Correlation coefficient test.

	Total Number of Detections per Patient	M (±SD)	Pearson Correlation	*p*-Value
Culture	0–2	0.579 (±0.587)	0.287	0.001
qPCR	0–8	2.244 (±1.796)		

**Table 7:** Resistance gene prevalence.

Antibiotic	Mechanism of Resistance	Resistance Gene	N (%) *
Carbapenem	Carbapenemase-hydrolyzing enzymes	*oxa-23*	38/65 (58.46%)
		*oxa-58*	38/65 (58.46%)
		*oxa-48*	38/65 (58.46%)
		*oxa-51*	22/65 (33.85%)
		*Imp*	2/65 (3.08%)
		*kpc*	-
		*ndm*	39/65 (60%)
		*vim*	4/65 (6.15%)
Vancomycin	Synthesis of modified peptidoglycan precursors with reduced binding affinity	*vanA*/*B*	1/5 (20%)
Quinolone	Synthesis of barrier protein for DNA gyrase	*qnr*	31/64 (48.44%)
Aminoglycoside β-lactam Cephalosporin Quinolone Trimethoprim–sulfamethoxazole	ESBL-hydrolyzing enzymes	*CTX-M*	26/64 (40.63%)
Methicillin	Encoding penicillin-binding protein 2a (PBP2a), which has a low affinity for beta-lactams	*mecA*/*mecC*	32/64 (50%)

* represents valid percentages.

**Table 8:** Comparison between culture sensitivity results and qPCR resistance gene detection.

Antibiotic (N) *	Culture		Resistance Genes	qPCR		*p*-Value (Pearson Chi-Square Test)
	Resistance N (%)	Sensitive N (%)		Detected N (%) **	Not detected N (%) **	
Imipenem (47)	36/47 (76.60%)	12/47 (23.40%)	o*xa-23*	27/36 (75%)	9/36 (25%)	0.530
			o*xa-58*	27/36 (75%)	9/36 (25%)	0.530
			*oxa-48*	32/36 (88.89%)	4/36 (11.11%)	0.004
			o*xa-51*	19/36 (52.78%)	17/34 (47.22%)	0.029
			OXA mix ***	35/36 (97.22%)	1/36 (2.78%)	0.023
			*imp*	1/36 (2.78%)	35/36 (97.22%)	0.855
			*kpc*	-	36/36 (100%)	-
			*ndm*	29/36 (80.56%)	7/36 (19.44%)	0.671
			*vim*	3/36 (8.33%)	33/36 (91.67%)	0.613
			*CRE* mix ***	30/36 (83.33%)	6/36 (16.67%)	0.565
			Overall ***	36/36 (100%)	-	0.003
Meropenem (37)	31/37 (83.78%)	6/37 (16.22%)	o*xa-23*	24/31 (77.42%)	7/31 (22.58%)	0.747
			o*xa-58*	24/31 (77.42%)	7/31 (22.58%)	0.747
			*oxa-48*	26/31 (83.87%)	5/31 (16.13%)	0.065
			o*xa-51*	16/31 (51.61%)	15/31 (48.39%)	0.116
			OXA mix ***	30/31 (96.77%)	1/31 (3.23%)	0.183
			*imp*	-	32/32 (100%)	-
			*kpc*	-	32/32 (100%)	-
			*ndm*	24/31 (77.42%)	7/31 (22.58%)	0.747
			*vim*	1/31 (3.23%)	30/31 (96.77%)	0.656
			*CRE* mix ***	25/31 (80.65%)	6/31 (19.35%)	0.878
			Overall ***	31/31 (100%)	-	0.021
Ertapenem (29)	26/29 (89.66%)	3/29 (10.34%)	o*xa-23*	18/26 (69.23%)	8/26 (30.77%)	0.019
			o*xa-58*	18/26 (69.23%)	8/26 (30.77%)	0.019
			*oxa-48*	24/26 (92.31%)	2/26 (7.69%)	0.005
			o*xa-51*	13/26 (50%)	13/26 (50%)	0.099
			Oxa mix ***	26/26 (100%)	-	<0.001
			*imp*	1/26 (3.85%)	25/26 (96.15%)	0.730
			*kpc*	-	26/26 (100%)	-
			*ndm*	25/26 (96.15%)	1/26 (3.85%)	<0.001
			*vim*	2/26 (7.69%)	24/26 (92.31%)	0.619
			*CRE* mix ***	25/26 (96.15%)	1/26 (3.85%)	<0.001
			Overall ***	26/26 (100%)	-	<0.001
Levofloxacin (16)	14/16 (87.5%)	2/16 (12.5%)	*qnr*	5/14 (35.71%)	9/14 (64.29%)	0.696
			*CTX-M*	5/14 (35.71%)	9/14 (64.29%)	0.308
Ciprofloxacin (47)	35/47 (74.47%)	12/47 (25.53%)	*qnr*	24/35 (68.57%)	11/35 (31.43%)	0.248
			*CTX-M*	21/35 (60.00%)	14/35 (40.00%)	0.270
Ampicillin–sulbactam (13)	12/13 (92.31%)	1/13 (7.69%)	*CTX-M*	6/12 (50%)	6/12 (50%)	0.335
Amoxicillin–clavulanic acid (12)	11/12 (91.67%)	1/12 (8.31%)	*CTX-M*	8/11 (72.73%)	3/11 (27.27%)	0.546
Piperacillin–tazobactam (39)	30/39 (76.92%)	9/39 (23.08%)	*CTX-M*	17/30 (56.67%)	13/30 (43.33%)	0.404
Aztreonam (14)	8/14 (57.14%)	6/14 (42.86%)	*CTX-M*	3/8 (37.5%)	5/8 (62.5%)	0.872
Cefoxitin (19)	17/19 (89.47%)	2/19 (10.53%)	*CTX-M*	10/17 (58.82%)	7/17 (41.18%)	0.811
Cefotaxime (31)	27/31 (87.10)	4/31 (12.90%)	*CTX-M*	15/27 (55.56%)	12/27 (44.44%)	0.441
Ceftazidime (41)	33/41 (80.49%)	8/41 (19.51%)	*CTX-M*	18/33 (54.55%)	15/33 (45.45%)	0.817
Ceftazidime–avibactam (10)	7/10 (70%)	3/10 (30%)	*CTX-M*	5/7 (71.43%)	2/7 (28.57%)	0.665
Ceftriaxone (33)	31/33 (93.94%)	2/33 (6.06%)	*CTX-M*	19/31 (61.29%)	12/31 (38.71%)	0.751
Cefepime (48)	39/48 (81.25%)	9/48 (18.75%)	*CTX-M*	23/39 (58.97%)	16/39 (41.03%)	0.465
Trimethoprim–sulfamethoxazole (20)	8/20 (40%)	12/20 (60%)	*CTX-M*	3/8 (37.5%)	5/8 (62.5%)	0.199
Gentamicin (48)	30/48 (62.50%)	18/48 (37.50%)	*CTX-M*	18/30 (60%)	12/30 (40%)	0.570
Amikacin (46)	28/46 (60.87%)	18/46 (39.13%)	*CTX-M*	14/28 (50%)	14/28 (50%)	0.239

* represents total number of isolates with sensitivity results toward antibiotic and detected resistance gene. ** represents valid percent out of resistant isolates toward antibiotic. *** OXA mix represents the presence of any oxa resistance genes, including *oxa-23*, *oxa-48*, *oxa-58*, and *oxa-51*. CRE mix represents the presence of any CRE genes, including *imp*, *kpc*, *ndm*, and *vim*. Overall represents the presence of any carbapenem resistance genes.

**Table 9:** Inclusion and exclusion criteria.

Inclusion criteria
Gender: Men and Women
Diagnosis: Patients with a clinical diagnosis of hospital-acquired pneumonia (HAP), including ventilator-associated pneumonia (VAP), as confirmed by medical records
Exclusion criteria
Diagnosis: Patients with clinical backgrounds that do not match with the HAP diagnosis criteria
Samples: Samples that were insufficient or contaminated were excluded from the analysis

**Table 10:** Case report form (CRF) clinical data overview.

Demographics
Gender
Age
Weight
Body mass index
Comorbidities
Location (ICU or Ward)
Laboratory investigations
Temperature
Blood pressure, heart rate, and respiratory rate
Biochemistry parameters including potassium, sodium, and creatinine
Hematology parameters including hemoglobin, hematocrit, and white blood cell count
Acid and base balance parameters including PH, PCO_2_, PO_2_, HCO_3_, and O_2_ saturation.
Inflammation parameters including CRP and ESR
Risk factors for HAP and/or VAP
Intubation and mechanical ventilation (including duration, in days, of oxygen therapy or mechanical ventilation)
Aspiration
Tube feedings
Oral/dental disease, poor oral hygiene, use of antibiotics in the last three months, use of oral antiseptics, poor infection control measures
Hyperglycemia
Supine positioning of the patient
Stroke or other neurologic disorders, seizures
Alcoholism
Gastroesophageal reflux disease (GERD)
Initial Findings
Symptoms onset (date and time)
Pleuritic chest pain
Shortness of breath (SOB), dyspnea, tachypnoea
Cough
Sputum production
Hemoptysis or rust-colored sputum
Muscular or joint pain, headache
Nausea and vomiting
Loss of appetite
Hypotension (BP < 90 mmHg)
Tachycardia (HR > 100 beats/min)
Altered breath sounds or localized rales
Chest radiography (dense lobar or segmental infiltrate)
Microbiological Diagnosis
Microbiology sputum culture and susceptibility results
Antibiotic used
Antibiotic treatment (empirical, initial, and next) with detailed dosage regimen and duration.
Outcome
Outcome of the patient, categorized as improved, partially improved, did not improve, worsening, or death
Time to clinical improvement, if applicable.

**Table 11:** LRB Oligo Mixes.

LRB Oligo Mix	Specific Nucleic Acid Amplification and Detection
LRB Oligo Mix 1	6-Carboxyfluorescein (FAM): *Streptococcus pyogenes* Hexachloro-6-carboxyfluorescein (HEX): Human Internal Control (IC) 6-Carboxy-X-rhodamine (ROX): *Enterobacteriaceae*
LRB Oligo Mix 2	FAM: *Haemophilus influenzae* ROX: *Mycoplasma pneumoniae*
LRB Oligo Mix 3	FAM: *Pseudomonas aeruginosa* ROX: *Streptococcus agalactiae* Indodicarbocyanine (CY5): *Escherichia coli*
LRB Oligo Mix 4	FAM: *Proteus* spp. HEX: *Serratia marcescens* ROX: *Klebsiella pneumoniae* CY5: *Acinetobacter calcoaceticus-baumannii* complex
LRB Oligo Mix 5	FAM: *Legionella pneumophila* HEX: *Klebsiella aerogenes* ROX: *Enterobacter cloacae* complex CY5: *Streptococcus pneumoniae*
LRB Oligo Mix 6	FAM: *Staphylococcus aureus* ROX: *Klebsiella oxytoca* CY5: *Moraxella catarrhalis*

**Table 12:** qPCR program protocol details.

Step	Cycle	Temperature	Duration
Enzyme Activation	1 cycle	52 °C	3 min
Pre-Incubation	1 cycle	95 °C	10 s
Denaturation		95 °C	1 s
Annealing and Extension	40 cycles	95 °C	1 s
		55 °C	15 s
Detection (Reading)		(FAM-Green) (HEX-Yellow)	(ROX-Orange) (CY5-Red)

**Table 13:** Resistance gene detection Oligo Mixes.

Urinary Tract Antibiotic Resistance Kit		Carbapenem Resistance Kit		Vancomycin Resistance Kit	
Oligo Mix	Intended Use	Oligo Mix	Intended Use	Oligo Mix	Intended Use
UTABR Oligo Mix 2		*CRE* Oligo Mix	FAM: *KPC*		
	FAM: *qnr*—Quinolone resistance		HEX: *NDM*	VRE Oligo Mix	FAM: *vanA*/*vanB*
	ROX: *vanB*—Vancomycin resistance		ROX: *VIM*		HEX: Human (Internal Control)
	CY5: *OXA-48*—Carbapenem resistance		CY5: *IMP*		
UTABR Oligo Mix 3		OXA Oligo Mix			
	FAM: *mecA*/*mecC*—Methicillin resistance		FAM: *OXA-51*		
	ROX: *CTX-M ESBL*		HEX: Human (Internal Control)		
			ROX: *OXA-23*/*OXA-58*		
			CY5: *OXA-48*		

## Data Availability

The data that support the findings of this study are available from the corresponding author upon reasonable request. Restrictions apply to the availability of these data due to privacy, ethical, and institutional policies.

## Abbreviations

The following abbreviations are used in this manuscript:

ALI	Acute Lung Injury
AMP	Antimicrobial Peptides
AP	Aspiration Pneumonia
ARDS	Acute Respiratory distress
BALF	Bronchoalveolar Lavage Fluid
CAP	Community-Acquired Pneumonia
CDC	Centers for Disease Control and Prevention
CRE	Carbapenem Resistance *Enterobacteriaceae*
CY5	Indodicarbocyanine
dNTPs	Deoxynucleotide Triphosphates
DNA	Deoxyribonucleic Acid
ESBL	Extended-Spectrum β-lactamases
ESR	Erythrocyte Sedimentation Rate
FAM	6-Carboxyfluorescein
GERD	Gastroesophageal Reflux Disease
GNB	Gram-Negative Bacteria
HAP	Hospital-Acquired Pneumonia
HEX	Hexachloro-6-Carboxyfluorescein
ICU	Intensive Care Unit
JASP	Jeffreys’s Amazing Statistics Program
LRB	Lower Respiratory Bacteria
MDR	Multi-Drug Resistance
MDR-GNB	Multi-drug-Resistant Gram-Negative Bacteria
MRSA	Methicillin-Resistant *Staphylococcus aureus*
NTC	No-Template Control
Oligo	Oligonucleotide
PBP2a	Penicillin-Binding Protein 2a
PDR	Pan-Drug-Resistant
PCR	Polymerase Chain Reaction
qPCR	Quantitative Polymerase Chain Reaction
RNA	Ribonucleic Acid
ROX	6-Carboxy-X-Rhodamine
SOB	Shortness Of Breath
SPSS	Statistical Package for the Social Sciences
UTABR	Urinary Tract Antibiotic Resistance
VAP	Ventilator-Associated Pneumonia
VRE	Vancomycin-Resistant Enterococci
